# Effect of hypertriglyceridemia on left ventricular global longitudinal strain in patients with coronary heart disease in Jilin Province, China: a cross-sectional study

**DOI:** 10.3389/fcvm.2023.1193971

**Published:** 2023-06-27

**Authors:** Lin Na, Wenjing Cui, Xinqi Li, Jing Chang, Xin Xue

**Affiliations:** ^1^ Department of Cardiology, The Second Hospital of Jilin University, Changchun City, China; ^2^ Clinical Laboratory, The Second Hospital of Jilin University, Changchun City, China

**Keywords:** coronary heart disease, hypertriglyceridemia, myocardial strain, cardiac function, speckle tracking

## Abstract

**Aims:**

Using speckle tracking technology to investigate the effect of hypertriglyceridemia on the global longitudinal strain(GLS) of the left ventricle in patients with coronary heart disease in the early stage, and to explore the value of myocardial strain in early identification of cardiac dysfunction in patients with coronary heart disease in the pre-heart failure stage.

**Methods:**

A cross-sectional study of 138 participants was conducted in Jilin Province, China. Basic clinical, biochemical, and echocardiographic data were obtained for all patients. Myocardial strain parameters were compared between the hypertriglyceridemia and normal triglyceride level groups and the effect of hypertriglyceridemia on early left ventricular global longitudinal strain impairment in coronary heart disease patients was evaluated.

**Results:**

The overall longitudinal strain of the left ventricle was smaller in the hypertriglyceridemia group than in the normal triglyceride group. After the multivariate Logistic regression model adjusting for the influence of confounding factors, the results remained stable.

**Conclusions:**

The risk of impairment of global longitudinal strain of the left ventricle in patients with coronary heart disease is positively correlated with triglyceride levels, and hypertriglyceridemia maybe an independent risk factor affecting early cardiac dysfunction in the pre-heart failure stage of patients with coronary heart disease.

## Introduction

Despite revascularization, coronary heart disease patients remain at a high risk for reduced ejection fraction and heart failure many years after the onset (1–5). Heart failure is a common causes of hospitalization among coronary heart disease patients (6, 7). Early interventions for the associated risk factors are crucial (4). Previous studies have reported that diabetes (8), hypertension (9), obesity (10), and multi-vessel disease are all independent risk factors for cardiac insufficiency in the late stages of coronary heart disease. It has also been reported recently that coronary heart disease patients with hypertriglyceridemia may be at a higher risk for new cardiac events (11, 12). However, it is unknown whether hypertriglyceridemia alters the myocardial structure in coronary heart disease patients in the early stages of heart failure.

Although left ventricular ejection fraction (LVEF) is commonly used to evaluate left ventricular systolic function, it has various limitations. It has low sensitivity for local myocardial damage (13), does not accurately reflect left ventricular myocardium function (14, 15), and cannot detect early myocardial dysfunction (16). Myocardial strain has greater sensitivity and accuracy for cardiac function and can assess the degree of myocardial deformation during the cardiac cycle, detect early left ventricular myocardial changes, and predict LVEF changes (17–19).

We used speckle tracking technology to explore the early effects of hypertriglyceridemia on left ventricular myocardial structure and function in coronary heart disease patients and to provide clinical evidence for myocardial remodeling caused by hypertriglyceridemia in the pre-heart failure stage.

## Methods

### Study population

We conducted a cross-sectional study in Jilin Province, China, between December 2021 and February 2022. A total of 138 coronary heart disease patients were recruited from the Department of Cardiovascular Medicine, Second Hospital of Jilin University. The diagnostic criteria for coronary heart disease was based on the American College of Cardiology and American Heart Association guidelines, i.e., stenosis > 50% in at least one coronary artery confirmed by coronary angiography or computed tomography (20). Furthermore, all patients included in the study presented symptoms of angina pectoris and were admitted with varying degrees of symptom severity. Furthermore, all patients included in the study presented symptoms of angina pectoris and were admitted with varying degrees of symptom severity. Furthermore, all patients included in the study presented symptoms of angina pectoris and were admitted with varying degrees of symptom severity. Exclusion criteria were symptoms and signs of heart failure and/or ejection fraction ≤ 50%, arrhythmias affecting cardiac function, congenital or secondary cardiomyopathies, valvular and other structural heart diseases, and severe hepatic or renal insufficiency. Patients who had experienced coronary artery occlusion or previous acute myocardial infarction were excluded from undergoing coronary angiography or coronary CT. Before collecting data, all patients had not received any intervention, that is, the ultrasound data and laboratory indicators of the patients The collection was completed before coronary CT examination, coronary angiography and PCI. The results of myocardial injury markers (troponin) were negative in all patients, and the levels of myocardial enzymes were within the normal range. The eligible participants were divided into hypertriglyceridemia (triglyceride [TG] ≥ 1.7 mmol/L, *n* = 60) and non-hypertriglyceridemia (TG < 1.7 mmol/L, *n* = 42) groups according to the American Endocrine Association clinical practice guidelines for hypertriglyceridemia (21). All participants signed informed consent before voluntary participation. The study complied with the Declaration of Helsinki and was approved by the Ethics Committee of the Second Hospital of Jilin University.

### Demographic and anthropometric characteristics

We recorded the gender, age, resting heart rate, and complications, such as hypertension and hyperglycemia, for all participants. Height and weight were measured twice with the participants barefoot and in light clothing; the average of the two values was then calculated. Body mass index (BMI) was calculated by dividing the weight (kg) by height (m) squared.

### Biochemical measurements

Blood samples were obtained from all participants and the following laboratory tests were performed: serum TG, serum high density lipoprotein (HDL-C), serum low density lipoprotein (LDL-C), serum total cholesterol, fasting glucose, glycosylated hemoglobin (HbA1c%), and estimated glomerular filtration rate (eGFR).

### Coronary GENSINI scores

The GENSINI score was calculated based on the participants' coronary angiography (22). Based on the part with the most severe stenosis, 1 point is scored for stenosis diameter< 25%, 2 points for 25%–49%, 4 points for 50%–74%, 8 points for 75%–89%, 16 points for 90%–98%, and 32 points for diameter≥ 99%. The above scores are multiplied by the corresponding coefficients of the coronary branches (left main branch: 5; proximal left anterior descending branch: 2.5, middle segment: 1.5, distal segment: 1; first diagonal branch: 1, second diagonal branch: 0.5; proximal left circumflex branch: 2.5, distal segment and posterior descending branch: 1, posterior collateral branch: 0.5, and proximal, middle, distal, or posterior descending branches of the right coronary artery: (1). The sum of the scores for all affected branches is the total score for a patient.

### Echocardiographic parameters

The EPIQ7C color Doppler ultrasound diagnostic instrument (Philips, Amsterdam, Netherlands) was used for the examination with a X5-1 cardiac probe at a probe frequency of 1.0–5.0 MHz. The measurements were repeated twice by an experienced cardiac sonographer in accordance with the American Society of Echocardiography criteria (23).

a. **Conventional echocardiography**
  The routine cardiac parameters were calculated as the average of three consecutive cardiac cycles. All participants were in sinus rhythm at the time of echocardiography. LVEF, left ventricular end-systolic volume, and left ventricular end-diastolic volume were measured using the Simpson biplane method under hemodynamically stable conditions. Interventricular septal thickness was measured using M-mode ultrasound. Pulse wave Doppler was used to measure the forward flow velocity of the mitral valve E-peak (early diastole) and A-peak (late diastole), and the E/A ratio was calculated.
b. **Two-dimensional speckle tracking echocardiography analysis**
  All participants were instructed to hold their breath during image acquisition to obtain high-quality images. While acquiring images, an electrocardiograph was connected to track the left ventricle membrane from the parasternal long-axis and short-axis views and apical two-chamber (2C), three-chamber (3C), and four-chamber (4C) views. Two-dimensional echocardiographic images of 3–5 complete cardiac cycles were obtained. The images were input into the Qlab13.0 workstation(Philips Healthcare, Andover, MA, USA) to automatically calculate the peak global longitudinal strain (GLS) of the left ventricular global systolic period. The measurements were repeated thrice for each participant and the average was calculated.

### Statistical analysis

All statistical analyses were performed using R version 3.3.2 (http://www.R-project.org; The R Foundation for Statistical Computing, Vienna, Austria), Free Statistics software version 1.7.1, and SPSS Statistics version 25.0 (IBM Corp., Armonk, NY, USA), Some graphics were constructed using GraphPad Prism Version 8.0 for Windows (GraphPad Software Inc., San Diego, CA, USA). Normally distributed data on Kolmogorov-Smirov test were expressed as means ± standard deviation, and t test was used for comparisons between two groups. Data with skewed distributions were expressed as medians (interquartile range); the rank sum test was used to compare two groups. Count data were expressed as the number of cases (n) and percentages (%), and were compared using the chi-square test. Pearson and Spearman correlation analyses were used to assess correlations between factors. Multicollinearity between covariates was assessed using variance inflation factor and tolerance values.

We assessed the association between the TG levels and the risk of GLS impairment using a multivariate logistic proportional hazards regression model by calculating odds ratios (ORs) and 95% confidence intervals (CIs). Five models were used to adjust for potential confounders. Model 1 was the crude model. Model 2 was adjusted for age, sex and comorbidities including hypertension. Model 3 was further adjusted for the risk factors when added it to this model, changed the matched odds ratio by at least 10 percent in model 1 (TC, HR). Model 4 further adjusted the risk factors when in univariate analysis, their *P* value were less than 0.05 (eGFR, FPG). Model 5 was adjusted for the clinically meaningful Gensini scores.

In the subgroup analysis, we examined the relationship between GLS impairment and the TG levels according to age (≤ 65 years vs.> 65 years), sex (male vs. female), BMI (< 24 vs. 24–28 vs.> 28 kg/m^2^), hypertension (no vs. yes), diabetes (no vs. yes), and hypertriglyceridemia (no vs. yes). Interactions between subgroups were assessed using the likelihood ratio test.

Due to the limited research available on the correlation between ultrasound speckle tracking technology and blood lipid levels, we were unable to find relevant literature regarding the relationship between triglyceride levels and impaired global longitudinal strain (GLS). Our sample size calculations were based on sample sizes from similar studies (24, 25) and results from the pre-test. The sample size was calculated using PASS version 11 (NCSS Statistical Software). Based on the similar studies and the pre-test of the trial, the average GLS in the non-hypertriglyceridemia group was −16.9 ± 3.8%, while that in the hypertriglyceridemia group was −14.3 ± 4.2%. With a two-sided *α* of 0.05, power of 80%, and a ratio between the two groups of 3:2, at least 48 and 32 participants were required in the non-hypertriglyceridemia and hypertriglyceridemia groups, respectively.

## Results

**Figure 1:**
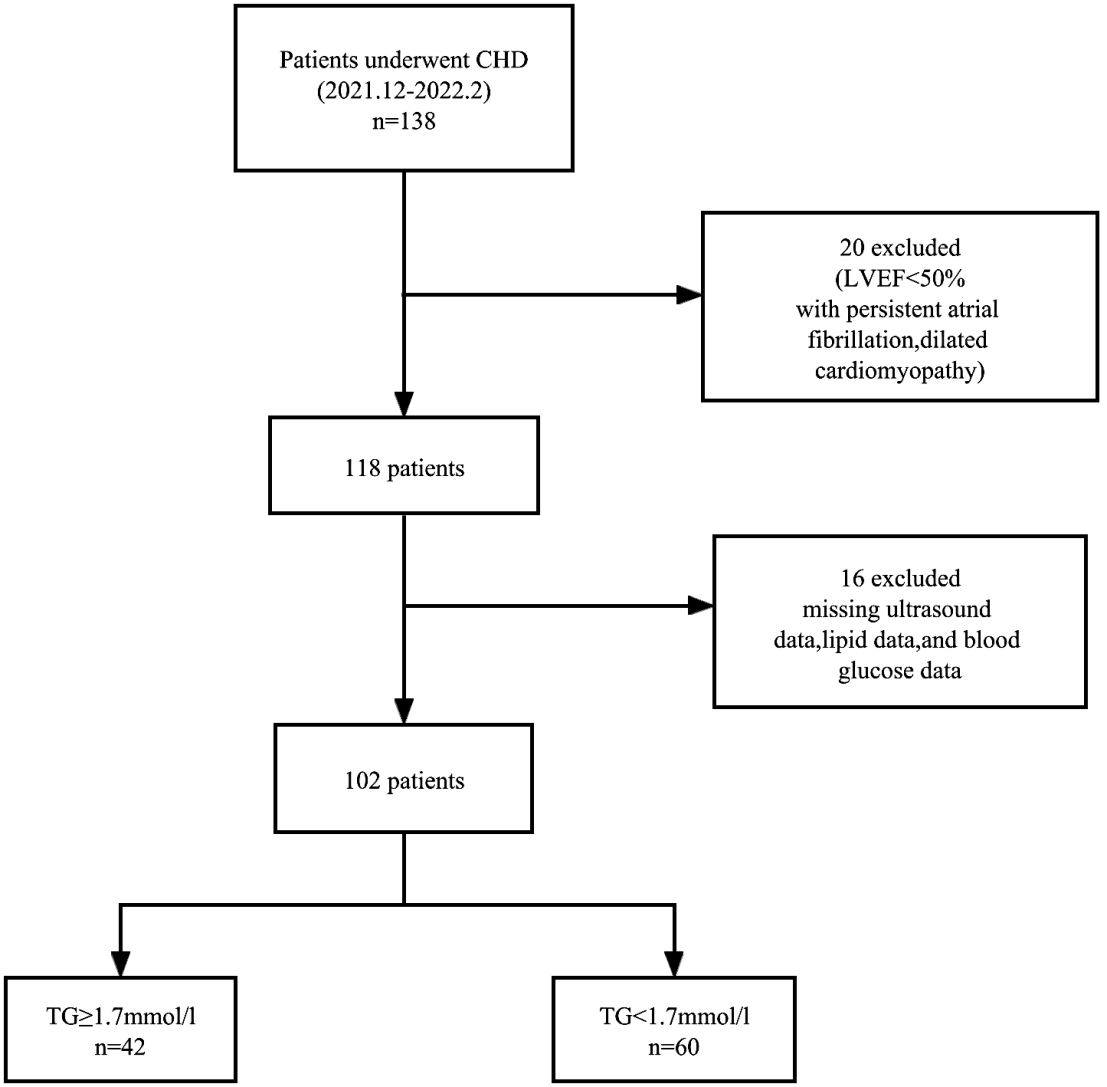
Flowchart of participant selection.

Of the 138 coronary heart disease patients initially evaluated, 14 with ejection fraction< 50%, five with missing ultrasound data, four with persistent atrial fibrillation, two with dilated cardiomyopathy, six with missing lipid data were excluded, and five with missing blood glucose data. A total of 102 patients met the inclusion criteria. Figure 1 is a flowchart of patient selection.

The 102 participants had a mean age of 62.9 years and included 59.8% males; 42 (41.2%) were diagnosed with hypertriglyceridemia. We used single regression imputation to fill in missing values on these covariates(missing values< 10%). Table 1 shows the basic clinical characteristics associated with hypertriglyceridemia. The resting heart rate in the hypertriglyceridemia group was significantly higher than in the non-hypertriglyceridemia group (*p* = 0.037). There were no significant differences between the two groups in terms of gender, age, BMI, and the frequency of diabetes and hypertension. TG and TC were significantly higher in the hypertriglyceridemia group compared to the non-hypertriglyceridemia group (*p* < 0.001). There were no significant differences in LDL-C, fasting blood glucose, HbA1c, and eGFR between the two groups. In addition, there was no significant difference in the GENSINI scores between the groups.

**Table 1:** Basic clinical characteristics for all participants.

Parameter	Control (*n* = 60)	High TG levels (*n* = 42)	*P*
Sex (M/F)	22 (36.7%)	19 (45.3%)	ns
Age (years)	63.2 ± 9.4	62.6 ± 9.6	ns
BMI (Kg/m^2^)	25.6 ± 3.3	25.7 ± 3.2	ns
HR (beat/min)	74.2 ± 9	78.6 ± 12	0.037
Hypertension *n* (%)	38 (63.3%)	23 (54.8%)	ns
Diabetes mellitus (%)	21 (35.0%)	14 (33.3%)	ns
Triglycerides (mmol/L)	1.17 (0.91–1.40)	2.31 (1.98–3.25)	<0.001
Total cholesterol (mmol/L)	3.76 (3.00–4.61)	4.86 (3.59–5.63)	<0.001
HDL-C (mmol/L)	1.01 (0.90–1.17)	0.88 (0.81–0.95)	0.001
LDL-C (mmol/L)	2.42 (1.67–3.07)	2.63 (1.86–3.63)	ns
FPG (mmol/L)	5.49 (4.89–6.92)	5.74 (4.69–7.09)	ns
HbA1c (%)	6.05 (5.70–7.33)	6.10 (5.65–7.50)	ns
eGFR (ml/min/1.73m^2^)	87.1 (72.4–97.2)	86.3 (73.2–98.2)	ns
Gensini score	35.5 (20.3–59.5)	40.0 (26.0–72.3)	ns

Data conforming to a normal distribution are shown as means ± SD, while those with skewed distributions are shown as medians (25th and 75th percentiles). Categorical variables are presented as absolute numbers (*N*) and percentages (%).

Ns, no significant; HR, heart rate; BMI, body mass index; Hb, hemoglobin; eGFR, estimated glomerular filtration rate; TC, total cholesterol; TG, triglyceride; HDL-C, high-density lipoprotein cholesterol; LDL-C, low-density lipoprotein cholesterol; HbA1c, glycosylated hemoglobin; FPG, fasting plasma glucose.

**Figure 2:**
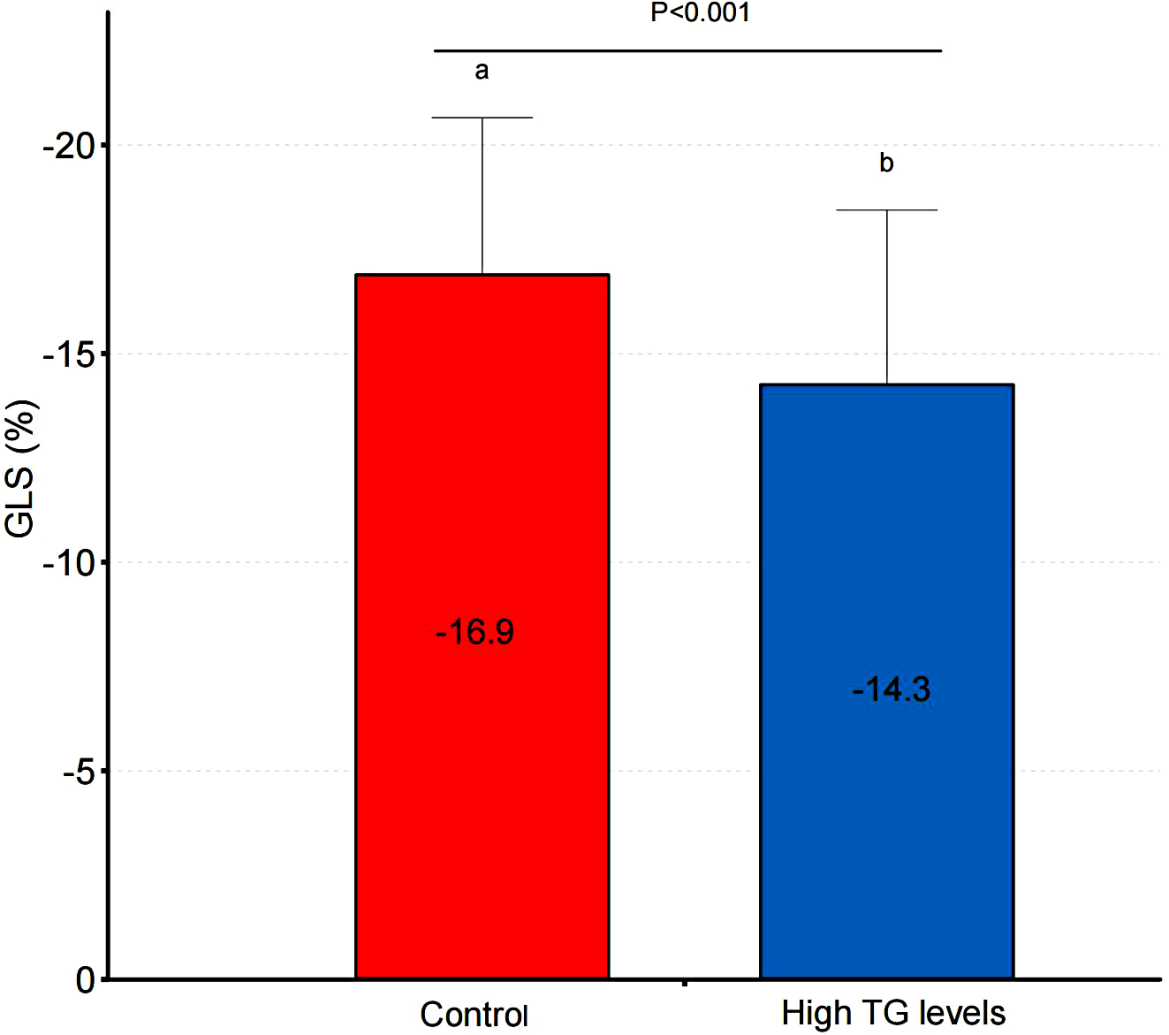
Comparison of GLS between high TG levels and control groups.

Table 2 shows the echocardiographic characteristics of the two groups. There were no statistically significant differences in routine echocardiographic parameters between the groups. GLS was significantly impaired in the high triglyceride group compared to the triglyceride control group (*p* < 0.05; Table 2, Figure 2).

**Table 2:** Echocardiographic characteristics for all participants.

Parameter	Control (*n* = 60)	High TG levels (*n* = 42)	*P*
Routine			
LVEF (%)	62.2 ± 4.7	62.4 ± 5.5	ns
LVESV	50.4 (37.6–63.5)	53.0 (38.8–62.0)	ns
LVEDV	129.5 (103.5–158.3)	138.0 (107.3–157.0)	ns
E	65.2 (51.4–77.5)	62.6 (51.3–67.3)	ns
A	91.2 ± 15.7	88.8 ± 14.8	ns
E/A	0.7 (0.6–0.8)	0.7 (0.6–0.8)	ns
e’	5.55 (4.71–6.61)	5.11 (4.30–6.31)	ns
E/e’	11.6 (9.28–13.75)	12.7 (9.90–14.20)	ns
Myocardial strain			
GLS(%)	−16.9 ± 3.8	−14.3 ± 4.2	0.001

Data with normal and skewed distributions are presented as means ± SD and medians (25th and 75th percentiles), respectively.

LVEF, left ventricular ejection fraction; LVESV, left ventricular end-systolic volume, left ventricular end-diastolic volume; E, early diastolic mitral valve peak E velocity.;A, peak late mitral inflow velocity; e, early diastolic mitral annular velocity; GLS, global longitudinal strain.

Table 3 shows the correlation between potential risk factors and GLS. The correlation analysis showed that TG, TC, fasting blood glucose, resting heart rate, and eGFR correlated with the GLS levels in coronary heart disease patients. Among these, TG levels and GLS were the most closely related, showing a moderate correlation (*r* = 0.376, *p* < 0.0001). Resting heart rate (*r* = 0.268, *p* = 0.006), TC (*r* = 0.226, *p* = 0.023), fasting blood glucose (*r* = 0.218, *p* = 0.028), and eGFR (*r* = −0.208, *P* = 0.036) were weakly correlated with GLS.

**Table 3:** Correlation analysis of potential risk factors for GLS.

Parameter	*r*	*P*
Age (years)	0.070	0.484
BMI (Kg/m^2^)	0.027	0.785
HR (beat/min)	0.268	0.006
Triglycerides (mmol/L)	0.376	<0.0,001
Total cholesterol (mmol/L)	0.226	0.023
HDL-C (mmol/L)	−0.139	0.165
LDL-C (mmol/L)	0.121	0.226
FPG (mmol/ll)	0.218	0.028
HbA1c (%)	0.143	0.153
eGFR (ml/min/1.73m^2^)	−0.208	0.036
Gensini score	0.181	0.169

*P* < 0.05 was considered significant.

BMI, body mass index; HR, heart rate; HDL-C, high-density lipoprotein cholesterol; LDL-C, low-density lipoprotein cholesterol; HbA1c, glycosylated hemoglobin; eGFR, estimated glomerular filtration rate.

**Figure 3:**
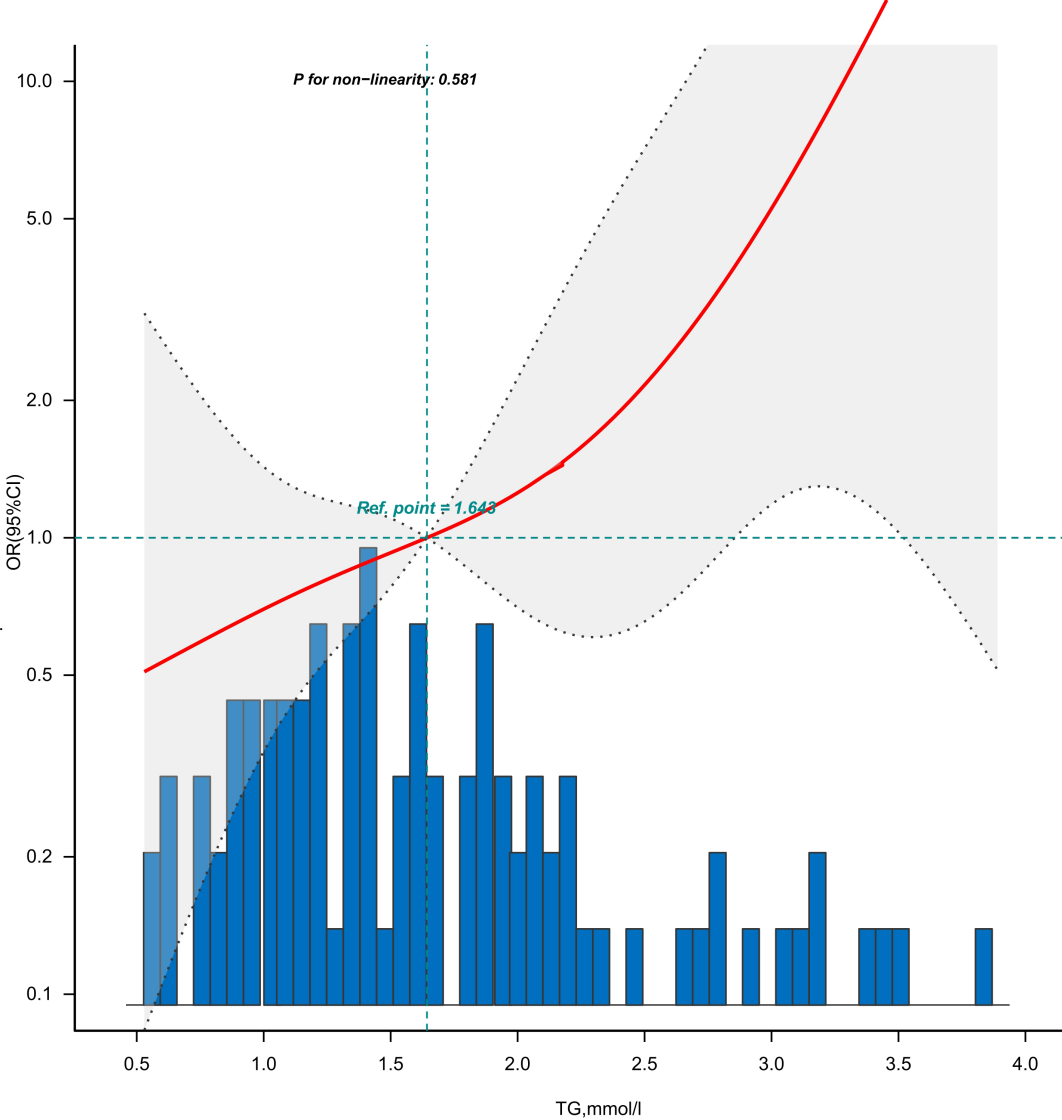
Dose-response relationship between the TG levels and adjusted risk of GLS impairment.

Figure 3 is the dose-response relationship between the TG levels and GLS impairment in coronary heart disease patients adjusted for Model 5. As shown in the image, the risk (incidence rate) of GLS impairment in patients with coronary heart disease showed an upward trend with the increase of TG levels.

Table 4 presents a multivariate logistic regression model of triglyceride levels and risk of GLS impairment in CHD patients. GLS impairment was defined as the cut-off point of −15.9% of the left ventricular GLS value (26). Both non-adjusted and multivariate adjusted models were applied to verify the stability of the results. Both non-adjusted and multivariate-adjusted models were used to verify the stability of the results. Variables for adjustment were selected on the basis of the following three criteria: (1) variables that, when added to the model, would change the matched OR by at least 10%; (2) variables with *P* < 0.05 in the univariate regression analysis; or (3) variables that were considered confounders based on existing literature and clinical judgment. Variables with strong multicollinearity were excluded. Based on the results of the tolerance value and variance inflation factor tests, the variables in the final model did not have multicollinearity. Adjusting for confounding factors did not change the association between the TG levels and impaired GLS (OR = 2.035, 95% CI 1.095–3.783 *P* = 0.025). For every 1 unit increase in the TG levels, the risk of GLS impairment increased 2.035-fold, indicating that hypertriglyceridemia maybe an independent risk factor for impaired left ventricular GLS in coronary heart disease patients.

**Table 4:** Multivariate analysis of parameters associated with low GLS patients.

Model	*P*	OR	95% CI
Model1	0.002	2.229	1.343–3.701
Model2	0.001	2.545	1.496–4.411
Model3	0.020	1.981	1.113–3.524
Model4	0.025	2.002	1.092–3.670
Model5	0.025	2.035	1.095–3.783

*P* < 0.05 is of significance.

Model 1: Crude model.

Model 2: Adjust for age, sex, hypertension.

Model 3: Adjust for Model 2 + HR,TC.

Model 4: Adjust for Model 3 + eGFR, FPG.

Model 5: Adjust for Model 4 + GENSINI score.

**Figure 4:**
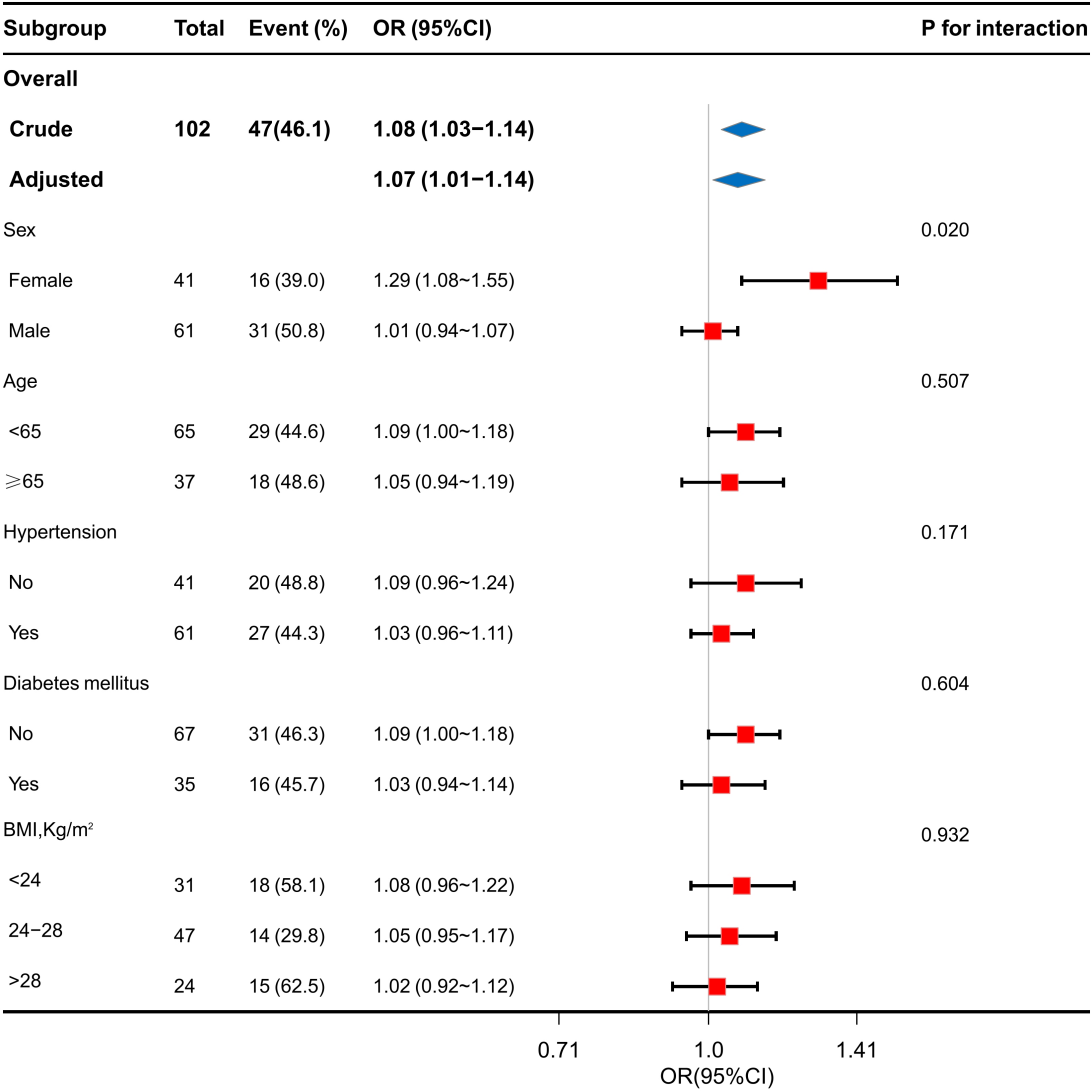
Stratification analysis on the association of TG levels with GLS impairment in coronary heart disease.

A forest plot of the subgroup analysis is shown in Figure 4. We performed a 10-fold transformation of triglyceride levels before subgroup analysis. Subgroup analysis showed a significant interaction between sex and the TG levels(*P* for interaction = 0.002). The association between the TG levels and the risk of GLS impairment was more significant in female patients than in male patients: OR 1.29 (95% CI 1.08–1.55) for female vs. 1.01 (95% CI 0.94–1.07) for male. The subgroup analysis showed that the association between the TG levels and GLS impairment was similar across patient subgroups stratified by age, gender, hypertension, diabetes mellitus, hypertriglyceridemia and BMI (*P* values for interaction > 0.05).

## Discussion

The present study found that CHD patients with hypertriglyceridemia had lower myocardial longitudinal strain levels. There was a correlation between TG levels and GLS reduction in coronary heart disease patients. After adjusting for the effects of confounding factors, such as fasting blood glucose, TC, resting heart rate, GENSINI score, and eGFR, using multivariate logistic regression analysis, the correlation still exists, indicating that hypertriglyceridemia maybe an independent risk factor for myocardial structural changes in pre-HF coronary heart disease patients.

The present study also found a potential correlation between high TG and high fasting blood glucose levels (*p* < 0.20); both of them caused myocardial structure and function impairments in pre-HF coronary heart disease patients (*p* < 0.05). High TG and high fasting blood glucose levels are the main components of metabolic syndrome (27), indicating a close relationship between metabolic abnormalities and early cardiac dysfunction in coronary heart disease patients (28, 29). Metabolic disturbances caused by high serum TG can contribute to subclinical myocardial injury and adverse cardiovascular outcomes (30, 31). Insulin resistance, the main cause of blood lipid and glucose metabolism disorders, promotes myocardial injury in coronary heart disease patients by promoting plaque formation and causing ventricular hypertrophy and abnormal diastolic function (32).

The mechanism by which hypertriglyceridemia causes early changes in the left ventricular structure in coronary heart disease patients is currently unknown. The relationship between hypertriglyceridemia and left ventricular function may be direct, indirect, or a combination. High serum TG levels directly cause myocardial steatosis and lipotoxic injury (33, 34). Specifically, high serum TG levels lead to increased myocardial TG content. This causes an imbalance between the uptake and utilization of fatty acids by cardiomyocytes and large amounts of lipids and intermediates accumulate in the myocardium. As a result, the epicardial fat pad expands, which has direct toxic effects on the subendocardial cavity and myocardium. This eventually leads to myocardial fibrosis and myocardial contractile dysfunction (33).

Meanwhile, hypertriglyceridemia also acts on the vasculature, leading to heart failure indirectly by mediating vascular remodeling and increasing the risk of ischemic cardiomyopathy (35). When free fatty acid levels are elevated, it can lead to oxidative stress and pro-inflammatory effects, which can aggravate myocardial ischemic injury by promoting the formation of vulnerable plaques, increasing the degree of coronary stenosis and calcification (36). The specific mechanism may be related to triglyceride-rich lipoproteins. The level of triglyceride actually reflects the level of TRL containing apolipoprotein B and very low-density lipoprotein in the circulation. Triglyceride may indirectly change the myocardial structure by affecting TRL, etc (37, 38). When serum TG levels are high, lipoprotein lipase activity decreases and triglyceride-rich lipoprotein increases (39). CETP catalyses the exchange of TGs for cholesterol between TRL and HDL and LDL particles HDL-C and LDL-C become smaller and denser, which allows easier entry into the arterial intima, promotes high expression of adhesion molecules, formation of foam cells, and eventually leads to a highly atherogenic state (40–42). Coronary arteriosclerosis reduces coronary blood flow; myocardial fibers in the endocardium are the most vulnerable to reduced coronary artery blood flow. Obstructed coronary arteries promote myocardial fibrosis and affect left ventricular myocardial function (43). To some extent, triglycerides are a marker that predicts early changes in myocardial structure in patients with coronary heart disease. The results of previous studies are less focused on patients with confirmed coronary heart disease. In agreement with our results, some scholars have found that high TG levels can cause early changes in the left ventricular systolic function in diabetic patients (44), which may be assessed early using GLS (43). The association between decreased GLS and high TG levels has also been reported (45).

Coronary heart disease patients with hypertriglyceridemia also had higher resting heart rates. A hypertriglyceridemic state reduces vagal excitability, which increases the basal heart rate (46, 47). Some scholars have found that an increase in the heart rate can lead to increased blood lipid levels (48, 49), and that there may be an interaction between hypertriglyceridemia and resting heart rate. High resting heart rate has also been shown to be associated with decreased early GLS in coronary heart disease patients; this remained true even after adjusting for confounding factors. Studies have shown that changes in the resting heart rate directly reflect the state of cardiac function and sympathetic tone, and that an increased resting heart rate can directly lead to myocardial damage (50–52). Hypertriglyceridemia may affect the level of longitudinal strain of left ventricular myocardium indirectly by affecting resting heart rate.

The present study did not find a correlation between BMI and early ventricular structural and functional changes in coronary heart disease patients. To the best of our knowledge, hypertriglyceridemia and obesity are closely related, and obesity may be an independent risk factor for reduced myocardial strain (53). A correlation between increased BMI and ventricular systolic function has been previously reported (54). Obesity can promote left ventricular volume increase and pressure overload, affecting myocardial structure and function (55, 56). In contrast with our study, a study in adolescents indicated that obesity may lead to early GLS impairment. This may be because of the greater average age in our study, population differences, and because the participants were all coronary heart disease patients. It has been reported that the overall longitudinal strain in the left ventricular myocardium may be used to assess subclinical myocardial dysfunction caused by hypertension (57, 58). This may be because our study population included coronary heart disease patients with relatively stable blood pressures on antihypertensive drugs, which may have led to insignificant myocardial damage.

The present study also found that in sex-stratified subgroups, the association between TG levels and the risk of impaired GLS in CHD patients was stronger in women than in men. Our findings are consistent with previous studies that have identified sex differences in triglyceride metabolism (59, 60). The mechanisms responsible for this difference are unclear and may arise from differences in the hormonal axis of the different sexes. Different sex hormones further affect myocardial structure and function by acting on lipid and glucose metabolism (61).

### Limitations

This study had some limitations. First, as this was a cross-sectional study with a small sample size, no causal relationships could be established. Unmeasured potential confounding factors may exist when assessing the effect of an increased TG levels on the GLS of the left ventricle in patients with coronary heart disease; however, the results of our sensitivity analysis showed that the results were stable across different subgroups. Meanwhile, this study focused on the effects of hypertriglyceridemia on left ventricular systolic function in coronary heart disease patients. In future research, stratified strain may be considered and left ventricular diastolic function may be further evaluated. In addition, this study used two-dimensional speckle tracking technology to obtain ultrasound images; image quality was limited by the conditions of the acoustic window, which may be difficult to view in the case of a low echo window.

## Conclusions

In patients with coronary heart disease, hypertriglyceridemia maybe cause early damage to the global longitudinal strain of the left ventricle (38). An optimal TG level maybe delay the progress of clinical heart failure in coronary heart disease patients. The control TG levels in coronary heart disease patients on the basis of existing statin therapy is crucial (62–66). In recent years, research on TG reduction has received an increasing amount of attention from scholars (67).

## Data Availability

The datasets presented in this article are not readily available because; data from this study may contain potentially or sensitive patient information. Requests to access the datasets should be directed to; xuexinsir@163.com.
